# Has the Financial Protection Been Materialized in Iranian Health System? Analyzing Household Income and Expenditure Survey 2003-2014 

**Published:** 2018-01-03

**Authors:** Hesam Ghiasvand, Alireza Olyaeemanesh, Reza Majdzadeh, Zhaleh Abdi, Mohammadreza Mobinizadeh

**Affiliations:** ^1^Social Determinants of Health Research Center, University of Social Welfare and Rehabilitation Sciences, Tehran, Iran; ^2^National Institute of Health Research and Health Equity Research Centre, Tehran University of Medical Sciences, Tehran, Iran; ^3^Community Based Participatory Research Centre and Knowledge Utilization Research Centre, Tehran University of Medical Sciences and Iran's National Institute of Health Research, Tehran, Iran; ^4^National Institute of Health Research, Tehran University of Medical Sciences, Tehran, Iran

**Keywords:** Health Equality, Fairness in financial contribution, Catastrophic health care payments, Financial protection

## Abstract

**Background:** The financial protection against catastrophic and impoverishing health expenditures is one of the main aspects of the universal health coverage. This study aimed to present a clear picture of the financial protection situation in Iran from 2003-2014.

**Study design:** This is an analytical study on secondary data of Statistical Center of Iran (SCI). The study has some policy implications for policy makers; therefore, it is an applied one.

**Methods:** Data related to the Iranian rural and urban household payments on health expenditures was obtained from annual surveys of the SCI. WHO researchers’ approach was used to calculate the Fairness of Financial Contribution Indicator (FFCI), the headcount and overshoot ratios of catastrophic and impoverishing health expenditures. A logistic regression was conducted to identify the determinants of probability of occurrence of catastrophic health expenditure among Iranian households in 2014.

**Results:** The mean of FFCI for rural and urban households was 0.854 (0.41) and 0.867 (0.32), respectively. The average headcount ratios of catastrophic and impoverishing health expenditures were 1.32% (0.24) and 0.33% for rural households and 1.4% (0.6) and 0.28% for urban households. Concerning rural households, the overshoot of catastrophic and impoverishing health expenditures was 14.94% and 7.22% (0.53); it was 15.59% (1.54) and 7.76% (0.52) for urban households.

**Conclusions:** No significant and considerable change was found in the headcount ratios of catastrophic and impoverishing health expenditure and in their overshoot or gap amounts. This suggested a lack of well-designed and effective schemes for materializing the financial protection in Iran.

## Introduction

Accessibility to health care services without any financial hardship and barriers is the concept of health equity. Providing health services and assuring their availability, accessibility and affordability is the main responsibility of governments^1,2^. Affordability of health services means protecting health users against catastrophic and impoverishing health expenditures. It is now considered one of the main goals of health systems^3^. Reducing the out-of-pocket payment, headcount ratios and gaps of catastrophic and impoverishing health expenditures are included in social welfare policies by many countries^4^. Despite the importance of financial protection, it is identified as a challenging goal especially in some developing countries^5^.

WHO emphasizes on the reduction of out-of-pocket and catastrophic health expenditure as a priority for governments. The Universal Health Coverage (UHC) is a main conceptual framework to assess the performance of health systems and financial protection being a focal aspect of it ^1,6^. Moreover, many countries have launched their plans to protect the households against catastrophic and impoverishing health expenditure through reducing the out-of-pocket expenses and redistributing the governmental health subsidiaries among different socioeconomic levels of the societies. Such plans have been developed and implemented by many countries ^7-9^.

In the reduction of out-of-pocket payments, catastrophic health expenditures have been considered in the national development documents ^10,11^. During the past years, however, it has been taken into huge account only in the recent Iranian Health Sector Evolution Plan (HSEP). This plan has been developed and implemented by Iranian Ministry of Health (in collaboration with other sectors including insurance organizations) since 2013. Heretofore, no formal documents were publicized to show reduction in the out-of-pocket, catastrophic, and impoverishing health expenditures although a 10% reduction was informally announced in OOP rate.

The rates and determinants of catastrophic and impoverishing health expenditures in national, regional, and local scales in Iran are studies. In the national level, the catastrophic health expenditure was about 2.46%, 3% and 2% for 2008, 2010 and 2013 respectively ^12,13^. In the provincial scale, about 22.2% of households in Kermanshah, western Iran experienced catastrophic health expenditures and that the fairness of financial contribution index (FFCI) was 22.2% and 0.57%, respectively ^14,15^.

Most of the financial protection studies in Iran have been conducted in a cross-sectional design and have not considered the trend of catastrophic and impoverishing health expenditures simultaneously, in a long-term view. In addition, the gap between catastrophic and impoverishing health expenditure, as a well-recognized tool to assess the current situation, was not taken into account. It helps policy makers and planners find the current situation of financial protection in Iran in a long-term and national representative study. According to WHO framework, analyzing the current situation is the first step in the development of an effective plan for financial protection ^16^.

This study aimed to estimate the fairness of financial contribution for health payments, the headcount and overshoot ratios of catastrophic and impoverishing health expenditures among Iranian rural and urban households in 2003-2014.

## Methods

### Data and Setting

The source of data was the Iranian Household Income-Expenditure Survey (IHIES) from 2002-2003 to 2013-2014. IHIES was conducted by Statistical Center of Iran (SCI) for one year, and data were obtained from direct interviews with the head of the household or any literate and informed member of the household.

IHIES included four modules: demographic and social characteristics of households, households’ expenditure for food, drinks, housing, entertainment and cultural affairs, education, health, transportation and communications and energy. The heads or informed adult members of the households are asked about the amount of money they spent on each of mentioned goods and services. The expenditure module included 13 chapters; the recall period was 4 wk for the first 12 chapters and 1 year for the 13^th^ chapter.

Chapters 6 and 13 represented the data about household health payments in terms of types of services and cares. Chapter 6 was about the outpatient services including visits, dental services, pharmaceutical services, medical consultations, etc., while chapter 13 was related to the hospitalization services such as types of surgeries. Totally, 93 codes were allocated to health in IHIES ^17^.

All data were recorded in the MS Access format and were transformed to the MS Excel format. The collected data was from the following variables:

Payments on different health services, food and non-food expenditures by household, gender of the head of the household, household size, house ownership status, age of the head of the household, rural/urban household residency, literacy status of the head of the household, the number of members under 5 or over 65, basic and complementary health insurance coverage and expenditure quartile of households.

### Population and Sampling

All Iranian households were the targeted population for SCI’s annually survey. The SCI performed a three-stage clustered sampling. In the first stage, the regions for accomplishing the census were selected and classified, and then each of these regions was divided into geographical blocks for both rural and urban households. Finally, some households were selected from every block. The SCI selected different sample sizes each year; the average number of households was 18632 and 17843 for rural and urban households respectively (2003-2004 to 2013-2014).

Fairness in financial contribution: The fairness in financial contribution among Iranian rural and urban households was calculated as follows:

(1) $$\text{FFCI=}1-\sqrt{\frac{\sum_{h=1}^{n}W_{h}\left|oopcpt_{h}-\;oopctp_{0}\right|}{\sum W_{h}}}$$

FFCI denotes the fairness in financial contribution to health care payments, Wh is the weight of each household in sample, oop shows the household out-of-pocket payment on health care, and ctp denotes the household capacity to pay (18). Thus, in this step the data about the weight of each household, the household payments on health expenditures, and capacity to pay was considered to calculate the FFCIs by rural and urban households in different years. The capacity to pay was defined based on the following steps ^18^.

CTP= Total Consumption Expenditure-Subsistence Expenditure (1)

Each household has its special CTP, consumption expenditure and subsistence expenditure.

And subsistence expenditure is:

(2) $$Subsistence\;Expenditure=\overset{55th}{\underset{45th}{\sum }}\frac{{\left(Equi.Food.Exp.\right)}_{45th-55th}}{W_{45th-55th}}$$

In equation (2), the subsistence expenditure is defined based on equivalent food expenditure for 45^th^ to 55^th^ expenditure percentiles (Equi.Food.Exp.)_45th-55th_. The equivalent food expenditure is derived from the equation (3):

(3) $${\left(Equi.Food.Exp.\right)}_{45th-55th}=\frac{{\left(Food\;Expenditure\right)}_{45th-55th}}{Equi.HHSize_{45th-55th}}$$

In the equation (3), Equi.HHSize45th-55th , is the equivalent household size for the households located in 45^th^- 55^th^ expenditure percentile. Finally, Equi.HHSize45th-55th, is derived from the equation (4):

Equi.HHSize_45th- 55th_= (Household Size)^0.56^

### The Catastrophic/Impoverishing Health Expenditures

WHO methodology was used to calculate the catastrophic/impoverishing health expenditures. It defined the catastrophic health expenditure as a situation in a household out-of-pocket payment on health equal to or greater than 40% of its capacity to pay ^18^. We considered the headcount ratios for catastrophic health expenditure and impoverishing as follows:

If out-of-pocket payment on health/capacity to pay ≥0.4; so the household was exposed to catastrophic health expenditure.

If the amount of out-of-pocket payment on health ≥ Households’ subsistence expenditure; so the household was exposed to impoverishing health expenditure.

The gap or overshoot of catastrophic/impoverishing health expenditure was another measure, which implied the intensity of catastrophic/impoverishing health expenditure. The overshoot revealed the distance of current ratio of out-of-pocket payment divided by the capacity to pay (with the considered thresholds). Thus, the equation (4) shows the overshoot

(4) $$O_{i}=\;E_{i}\left(\left(\frac{OOP_{i}}{CTP_{i}}\right)-\;Z\right)$$

In equation (4) the O_i_ is the overshoot measure for household i, OOPi is the amount of out-of-pocket payment, CTP_i_ is the capacity to pay of household i, and Z is the catastrophic threshold considered 0.4 in this study. For impoverishing health expenditure gap, the equation (5) was used:

(5) $$G_{poverty}=\frac{1}{N}\overset{N}{\underset{1}{\sum }}g_{i}\;and\;g_{i}=X_{i}-\;PL$$

In equation (5) the G_poverty_ is the impoverishing health expenditure gap, N shows the total number of samples, Xi denotes the household income/ total expenditure and PL is the poverty line. The poverty line equals the subsistence expenditure in this study ^19^.

### Determinants of Catastrophic Health Expenditures

A logistic regression with an “*enter* ” approach was conducted to analyze the association between likelihood of occurrence of catastrophic health expenditures and covariates. The regression was performed for the last year of the study (2013-2014). The covariate list was presented in the last part of the data and setting section. Data were analyzed using the Stata 14.

## Results

During the past 11 yr, the mean sample size was 18632 and 17843 for rural and urban households, respectively. About 52% of samples were rural households and 48% were urban households. During this period, 87% of the urban households had a male head and 85% of the head of the rural households was male. The mean household size was 3.85% and 3.63% for rural and urban households, respectively. The literacy rate of the head was about 69% in rural and 82.5% in urban households. About 84% of rural and 80% of urban heads were married; the divorce rate was about 6% and 7% in rural and urban households respectively. Iranian Basic Health Insurance Schemes covered about 96% of rural and 76% of urban households. Moreover, 29% of urban and 33% of rural households had members less than 5 yr of age and about 23% of rural and 17% of urban households had members 65 and more. In addition, the mean payment on health expenditures, as a share of total expenditures, was 9.3% and 8.5% for rural and urban households respectively.

The maximum range of FFCI was respectively 0.9 (0.043), and 0.866 (0.65) for urban and rural households in 2006-2007. The minimum range of FCCI was 0.833 (0.51) for urban households in 2005-2006 and 0.836 (0.36) for rural households in 2013-2014 (Table 1).

**Table 1:** Fairness in Financial Contribution Index (FFCI) for Iranian out-of-pocket payments on health services (Iran Rial)

**Year**	**Rural FFCI** **(95% CI)**	**Urban FFCI** **(95% CI)**
2003- [2004 (first season)]	0.854 (0.825, 0.873)	0.870 (0.857, 0.882)
2004- [2005 (first season)]	0.851 (0.836, 0.874)	0.873 (0.806, 0.894)
2005- [2006 (first season)]	0.862 (0.841, 0.883)	0.874 (0.831, 0.893)
2006- [2007 (first season)]	0.851 (0.820, 0.871)	0.833 (0.811, 0.854)
2007- [2008 (first season)]	0.866 (0.842, 0.882)	0.901 (0.886, 0.924)
2008- [2009 (first season)]	0.850 (0.842, 0.861)	0.871 (0.850, 0.894)
2009- [2010 (first season)]	0.850 (0.845, 0.872)	0.874 (0.857, 0.886)
2010- [2011 (first season)]	0.861 (0.845, 0.872)	0.871 (0.861, 0.882)
2011- [2012 (first season)]	0.853 (0.842, 0.860)	0.870 (0.858, 0.880)
2012- [2013 (first season)]	0.860 (0.801, 0.872)	0.852 (0.840, 0.858)
2013- [2014 (first season)]	0.836 (0.831, 0.856)	0.858 (0.750, 0.858)
Mean	0.854 (0.834, 0.861)	0.867 (0.856, 0.872)

The values and trend of headcount ratios of catastrophic and impoverishing health expenditures for Iranian rural and urban households were shown in (Table 2). The maximum amount of head counts of catastrophic health expenditures was 1.98% (0.65) for rural households in 2009-2010 and 1.94% (0.72) for urban households in 2010-2011. The minimum range of headcount ratios was 0.5% (0.11) and 0.48% (0.09) for rural and urban households respectively in 2013-2104. Concerning the headcount ratios of impoverishing health expenditures and headcount ratios, the maximum range was 1.03% (0.6) for urban households in 2003-2004 and 1.46% (0.54) for rural households in 2009-2010. The minimum range of headcount ratios of impoverishing health expenditures was respectively 0.031% (0.001) and 0.03% (0.001) for rural and urban households in 2013-2014.

**Table 2:** The headcount ratios (%) of catastrophic and impoverishing health expenditures in Iran

**Year**	**Rural**		**Urban**	
	**Catastrophic (95% CI)**	**Impoverishing (95% CI)**	**Catastrophic (95% CI)**	**Impoverishing (95% CI)**
2003- [2004 (first season)]	1.35 (1.30, 1.37)	0.85 (0.70, 0.90)	1.30 (1.25, 1.36)	0.87 (0.70, 0.90)
2004- [2005 (first season)]	1.29 (1.10, 1.82)	0.76 (0.70, 0.80)	1.04 (0.90, 2.20)	1.03 (0.90, 1.10)
2005- [2006 (first season)]	1.22 (1.00, 1.40)	1.14 (0.92, 1.30)	1.42 (1.30, 1.60)	0.82 (0.70, 0.90)
2006- [2007 (first season)]	1.80 (1.60, 1.90)	0.80 (0.70, 0.88)	1.20 (1.01, 1.30)	0.92 (0.80, 1.20)
2007- [2008 (first season)]	1.38 (1.24, 1.51)	0.90 (0.76, 1.10)	1.44 (1.27, 1.62)	0.83 (0.77, 0.85)
2008- [2009 (first season)]	1.78 (1.60, 1.91)	1.00 (0.80, 1.10)	1.50 (1.00, 1.70)	1.00 (0.70, 1.10)
2009- [2010 (first season)]	1.98 (1.80, 2.10)	1.46 (1.20, 1.90)	1.65 (1.50, 1.80)	1.02 (0.80, 1.10)
2010- [2011 (first season)]	1.00 (0.80, 1.20)	0.65 (0.20, 0.90)	1.94 (1.30, 2.60)	0.72 (0.60, 0.90)
2011- [2012 (first season)]	1.30 (0.90, 1.60)	0.02 (0.01, 0.05)	0.74 (0.70, 0.80)	0.05 (0.01, 0.08)
2012- [2013 (first season)]	0.87 (0.70, 1.00)	0.87 (0.60, 0.90)	0.66 (0.50, 0.83)	0.75 (0.60, 0.87)
2013- [2014 (first season)]	0.50 (0.40, 0.60)	0.03 (0.02, 0.04)	0.48 (0.40, 0.60)	0.03 (0.02, 0.04)
Mean	1.32 (1.30, 1.40)	0.33 (0.20, 0.38)	1.40 (1.30, 1.50)	0.28 (0.20, 0.35)

The overshoot of catastrophic and impoverishing health expenditure, as a measure of gap analysis, was presented through overshoot measures for both catastrophic and impoverishing health expenditures in table 3. The maximum amount of overshoot of catastrophic health expenditures was 19.74% (1.86) for rural households in 2009-2010 and 20% (1.9) for urban households in 2006-2007. The minimum range of overshoot of catastrophic health expenditures was 11.7% (1.01) and 11.45% (1.08) for rural and urban households respectively in 2013- 2014. The maximum range of overshoot of impoverishing health expenditures was 9.8% (1.04) for rural households in 2004-2005 and 14.23% (1.76) for urban households in 2005-2006. The minimum range of overshoot of impoverishing health expenditures was 3.8% (0.07) for rural households in 2012-2013 and 3.2% (0.024) for urban households in 2013-2014.

**Table 3:** The overshoot of catastrophic and impoverishing health expenditures in Iran

**Year**	**Rural**		**Urban**	
	**Catastrophic (95% CI)**	**Impoverishing (95% CI)**	**Catastrophic (95% CI)**	**Impoverishing (95% CI)**
2003- [2004 (first season)]	14.6 (14.1, 14.4)	7.9 (4.2, 12.4)	13.7 (13.0, 14.1)	5.8 (2.1, 9.8)
2004- [2005 (first season)]	13.7 (13.6, 13.8)	7.6 (5.4, 9.5)	16.7 (16.7, 16.8)	10.5 (8.5, 12.0)
2005- [2006 (first season)]	16.0 (15.3, 16.2)	9.8 (5.4, 13.0)	16.7 (16.6, 16.8)	10.1 (7.0, 14.0)
2006- [2007 (first season)]	13.2 (13.1, 13.3)	9.2 (5.3, 13.6)	18.0 (17.9, 18.1)	14.2 (10.3, 16.5)
2007- [2008 (first season)]	16.1 (16.1, 16.2)	8.8 (5.4, 11.0)	20.0 (19.9, 21.2)	12.0 (7.5, 15.4)
2008- [2009 (first season)]	15.6 (15.1, 16.2)	6.8 (3.4, 9.7)	17.2 (17.2, 17.3)	8.6 (5.0, 12.0)
2009- [2010 (first season)]	19.7 (19.7, 19.8)	8.6 (3.4, 11.0)	16.2 (15.8, 16.7)	7.2 (3.4, 10)
2010- [2011 (first season)]	18.7 (17.0, 19.4)	9.0 (6.4, 12.0)	17.0 (16.5, 18.4)	6.5 (3.2, 8.0)
2011- [2012 (first season)]	13.2 (12.0, 14.0)	4.8 (1.4, 8.6)	11.5 (10.0, 12.0)	4.2 (1.8, 9.5)
2012- [2013 (first season)]	11.9 (11.0, 12.2)	3.8 (0.9, 5.7)	12.9 (12.3, 13.6)	5.0 (1.2, 7.2)
2013- [2014 (first season)]	11.7 (8.6, 14.6)	3.1 (1.0, 7.4)	11.4 (11.4, 12.4)	3.2 (1.8, 6.5)
Mean	14.9 (13.7, 15.3)	7.2 (5.3, 8.5)	15.6 (14.8, 16.2)	7.7 (5.3, 9.3)

Living in rural regions, having literate heads, owning a house, living in a rental house and placing in higher total expenditures quartiles were significant determinants of exposure to catastrophic health expenditure. Of course, we considered the significance level at 10% (Table 4).

**Table 4:** The determinants of catastrophic health expenditure occurrence in Iran (2013-2014)

**Variables**	**Unadjusted Odd Ratio** **(95% CI)**	**Adjusted Odd Ratio** **(95% CI)**
Age square of the head of the household	0.99 (0.99, 1.00)	0.78 (0.42, 1.45)
Household size	1.00 (0.91, 1.09)	0.94 (0.77, 1.78)
Gender of Household’s Head	
Male	1.00	1.00
Female	0.96 (0.62, 1.50)	1.02 (0.51, 2.05)
Region of Residency		
Urban	1.00	1.00
Rural	1.01 (0.99, 1.02)	0.70 (0.46, 1.08)
Household’s Head Literacy Status	
Illiterate	1.00	1.00
Literate	0.94 (0.91, 1.05)	0.45 (0.21, 0.98)
Members under 5 yr of age	
No	1.00	1.00
Yes	1.23 (0.91, 1.66)	1.53 (0.87, 2.72)
Members over 65 yr of age	
No	1.00	1.00
Yes	1.10 (0.76, 1.60)	0.54 (0.22, 1.31)
Ownership of House		
Complete Ownership (non-apartment house)	1.00	1.00
Owning a flat	1.27 (0.72, 2.71)	2.94 (0.86, 9.98)
Rent	1.02 (0.86, 2.32)	0.50 (0.22, 1.14)
Mortgage	1.62 (1.04, 2.89)	0.36 (0.43, 2.92)
Organizational	1.23 (0.84, 1.75)	1.12 (0.39, 7.79)
Free	0.96 (0.85, 1.23)	0.98 (0.01, 6.54)
Having a basic Health Insurance Coverage	
No	1.00	1.00
Yes	1.01 (0.80, 1.51)	0.97 (0.59, 1.61)
Having a complementary Health Insurance Coverage	
No	1.00	1.00
Yes	1.22 (0.66, 2.24)	0.94 (0.43, 2.05)
Total Expenditure Quartiles	
1st	1.00	1.00
2nd	0.89 (0.16, 1.97)	1.84 (1.01, 3.34)
3rd	1.87 (1.03, 2.61)	6.22 (2.11, 18.41)
4th	3.87 (1.04, 5.87)	5.14 (3.42, 23.67)

## Discussion

Financial protection, as one of the most important goals in any health system, must be considered a prerequisite to reaching universal health coverage. This paper analyzed the trend of fairness in financial contribution to health payments, headcount, and overshoot of occurrence of catastrophic and impoverishing health expenditures and the determinants of exposure to the catastrophic health expenditures in 2013-2014. The mean fairness in financial contribution indicator was 0.854 (0.41) for rural households and 0.867 (0.32) for urban households.

The mean headcount ratios of catastrophic and impoverishing health expenditures were 1.32% (0.24) and 0.33% (0.006) in rural households and 1.4% (0.6) and 0.28% (0.001) in urban households. The mean overshoot of catastrophic and impoverishing health expenditures was 14.94% (1.12) and 7.22% (0.53), as well as 15.59% (1.54) and 7.76% (0.52) for rural and urban households respectively.

Considering the significance level of 10%, the significant predictors of exposure to catastrophic health expenditures included: having a flat, renting a house or a free house without paying any rent or mortgage, a literate head, living in rural regions and being placed in 2^nd^, 3^rd^ and 4^th^ quartiles of total consumption expenditure.

The headcount and overshoot ratios of catastrophic and impoverishing health expenditures did not show considerable differences between rural and urban households. In addition, their trend did not change notably over the given time. In fact, the Iranian governments did not have any significant achievements to reach the goals of national development plans.

At first glance, the FFCIs figures implied a relatively good situation in establishing fair financing mechanisms for health services in Iran although financing current situation is not satisfactory due to the national focus on improving equality in healthcare systems. In addition, if the trend of FFCIs was taken into account during the time of study, it implied that the states could not establish effective mechanisms to transfer the burden of financial contribution to richer groups. The FFCI was under 0.82 in 2009 for Georgia and 0.735 in 2001 for China^20-22^.

Several domestic studies have been conducted on the rate of FFCI, the catastrophic and impoverishing health expenditures rates and their determinants in Iranian national scales. In one study, the FFCI was calculated between 2003 and 2010 and was, on average, 0.841, and 0.827. Moreover, the rates of the catastrophic health expenditure varied from 2.3% to 3.1% in the mentioned years^12^. A range between 0.84 to 0.91 and 0.85 to 0.94 was calculated for FFCI for Iranian rural and urban households during different years in different studies ^22-24^. In this study, mean FFCI was in line with previous evidence and a little difference was observed between their results.

Nevertheless, the FFCI was about 0.57 in Kermanshah. The difference between that and the present study might be related to different sample sizes and the characteristics of samples (in the mentioned study, it included the residents of Maskan population in Kermanshah) ^15^.

The data management and manipulation seems not to be same in previous studies and in addition to most of them, the weights of households, as an influencing factor did not consider properly by researchers. Therefore, sometimes the results of this study may be not in accordance with previous studies.

Concerning the rate and determinants of catastrophic health expenditure, the rate of exposure to catastrophic health expenditure was about 2.1% in 2010^23^. Other studies showed a range between 2.8% and 5.4% for Iranian households in different years by SCI’s annual surveys^24-26^.

Results of the regression revealed notable points: firstly, households with better socio-economic status such as those who had houses and those who spent more consumption expenditures experienced more catastrophic health expenditure compared to poorer households. Moreover, rural residency had a protective effect on prevention of catastrophic health expenditure. At first glance, one may suppose these are not rational, but in a deeper consideration, the economic situation of Iran was not good in this year and the health sector faced challenging conditions due to sanctions and macroeconomic instability. In addition, the inflation level was very high and many drugs and medical technologies and equipment were not affordable for households; moreover, the health insurance and the government could not provide them without imposing financial hardship. Under these circumstances, only the households with higher levels of income and economic status, especially in urban regions, could take advantage of health services.

The main determinants of exposure to catastrophic health expenditures included being placed in lower quintiles of expenditure, living in rural regions, having members younger than 5 yr of age, having the health insurance coverage and households with a male as the head ^27^. Another study on Colombian households revealed that households with children or old members, living in rural areas, households with more members and not being insured by healthcare systems were determinants of occurrence of catastrophic health expenditure^28^.

The determinants of catastrophic health expenditure were living in rural regions, having members older than 65 yr of age, illiteracy of the heads, unemployment of the heads, having unemployed members, households with higher levels of income and households with larger equivalent household size (larger than the average size of the community) ^14,23^.

In fact, several studies have been carried out using the data adopted from SCI; the Center is one of the most valid and reliable sources for conducting research in social welfare field. The SCI survey was not a specific one for the health, and the questions about the health expenditures had not been standardized according to WHO and World Bank advice. The main part of questions was dedicated to outpatient services, but the questions about the in-patient services were limited. Concerning outpatient services, there were some considerable variations such as practitioners’ visits and consultations, diagnostic services, and drugs; there was no accurate classification of services. The SCI database was not linked with other health bodies such as Ministry of Health and Health Insurers. It caused the figures about the payments in hospitals to be inaccurate. In addition, some services such as traditional medicine, herbal drugs, cosmetic goods and plastic surgery were included in the survey. The unavailability of the data was another pitfall of this survey. Therefore, the researchers faced a survey with low levels of standardization process, and some of them manipulated the data and variables based on their arbitrations. This is one of the main causes of differences in the headcount ratios of catastrophic and impoverishing health expenditures.

Besides methodological challenges, one cannot ignore the role of Iran’s policies and plans in the current situation. The government could not develop a comprehensive and detailed profile of health and socio-economic status of all Iranian households. This profile should encompass the Iranian household demographics, health and socioeconomic information^21,22^. Moreover, Iran cannot tackle financial protection effectively and thus cannot identify and protect the vulnerable groups against catastrophic and impoverishing health expenditures.

The macroeconomic instability and the government’s budget deficit reduced the budgets allocated to health authorities, and the contribution of Iranian health insurers was not considered in health care financing. Under such conditions, the government must revise some mismanagement and prevent the waste of financial resources. According to WHO’s report in 2010, one of the best mechanisms to provide new financial resources for health systems is developing strategic purchasing, clinical practice guidelines, new and effective payment /reimbursement mechanisms, etc. ^4^.

Thus, the minimum level of headcount and overshoot ratios of catastrophic/impoverishing health expenditures in 2013-2014 showed that the government was unsuccessful in reducing the catastrophic/impoverishing health expenditures.

The mean out-of-pocket expenditure, as a percent of total health expenditure, has been about 52% over the past 15 yr; the government has launched the Health Sector Evolution Plan (since 2013) whose first goal is to protect Iranian households financially. At the time of the research in Dec 2015, however, there was no formal evidence about the performance of Iranian government to achieve this goal.

This plan targeted the hospitals affiliated to Iranian Ministry of Health and other public authorities and was very active in protecting households hospitalized in these hospitals. Nevertheless, the main part of outpatient services such as dental care, medical imaging, and laboratory tests were provided by non-public providers with market prices. Hence, this plan seemed to face limitations for reducing the out-of-pocket payments and catastrophic and impoverishing health expenditures.

The financial resources of health system in Iran are not integrated well and it leads to partial risk pooling and dispersing financial protection pattern. Now, the population does not benefit from financial resources based on their socioeconomic status and their health profile. Under this condition, the unprivileged groups and poor people cannot utilize the health services about their capacity to pay and meet the needs ^22^.

Besides the Iranian Health Sector Evolution Plan, a national plan called Iranian Universal Health Insurance Plan has been launched. This plan is implemented to cover Iranian population totally. In 2005, Iranian government implemented another national health insurance plan to cover the vulnerable people in rural regions and cities with a population less than 20000. These two major health care financing plans are managed by Iranian Health Insurance Organization (IHIO). These are promising activities that the Iranian State has launched; however, their sustainability is not clear.

## Conclusions

Achieving financial protection against catastrophic and impoverishing health expenditure and fairness in financial contribution of health users was materialized in Iran from 2003 to 2014. Developing effective financing schemes through extending the health insurance coverage and increasing the share of government in paying the health expenditures has been recognized by Iranian policymakers. In response to this requirement, the Iranian government has implemented the Health Sector Evolution Plan. However, many experts criticize the effectiveness of HSEP and the economic conditions of the country do not let the government implement it properly. Thus in terms of sustainability of the resources and directing the resources towards most deprived groups, seems that providing the health subsidies to middle and lower economic groups in an appropriate method based on their abilities to pay and on their health needs require a prompt action by the government.

The financial protection requires a specific policy-making and managerial entity to lead other bodies and to establish the intersectional coordination. Thus, Iranian health system needs two national plans to protect the population against catastrophic health expenditures: firstly, a plan to protect the vulnerable and poor classes, and secondly, designing a benefit package to include diseases/illness and medical services with catastrophic/ impoverishing outcomes.

## Ethical Considerations

All data was obtained from SCI using the formal permissions.

## Acknowledgements

The authors greatly appreciate Mr. Hassan Varmazyar due to his cooperation in cleaning the raw data.

## Conflict of interest statement

The authors declare that there is no conflict of interest.

## Funding

This study was supported by I.R. Iran’s National Institute of Health Research, Tehran University of Medical Sciences as a part of the project “Financial protection against catastrophic and Impoverishing Health Expenditure in I.R.I Iran”.

## Highlights

- Over the considered time span, no significant and considerable change was found in the catastrophic and impoverishing health expenditure indexes.
- A plan to protect the vulnerable and poor classes must be designed.
- A benefits package for including services with catastrophic/impoverishing outcomes must be designed.
- Directing the health subsidies to middle and lower economic groups in an appropriate method must be implemented.
